# Cranial bone microarchitecture in a mouse model for syndromic craniosynostosis

**DOI:** 10.1111/joa.14121

**Published:** 2024-08-02

**Authors:** Sara Ajami, Zoe Van den Dam, Julia Hut, Dawn Savery, Milton Chin, Maarten Koudstaal, Miranda Steacy, Alessandra Carriero, Andrew Pitsillides, Y.‐M. Chang, Christoph Rau, Shashidhara Marathe, David Dunaway, Noor Ul Owase Jeelani, Silvia Schievano, Erwin Pauws, Alessandro Borghi

**Affiliations:** ^1^ UCL Great Ormond Street Institute of Child Health University College London London UK; ^2^ Craniofacial Unit, Great Ormond Street Hospital London UK; ^3^ Oral and Maxillofacial Department, Erasmus MC Rotterdam The Netherlands; ^4^ Department of Biomedical Engineering The City College of New York New York NY USA; ^5^ Comparative Biomedical Sciences, The Royal Veterinary College Royal College Street London UK; ^6^ Diamond Light Source, Harwell Science and Innovation Campus Didcot UK; ^7^ Department of Engineering Durham University Durham UK

**Keywords:** bone, craniofacial, craniosynostosis, Crouzon, FGFR2

## Abstract

Crouzon syndrome is a congenital craniofacial disorder caused by mutations in the Fibroblast Growth Factor Receptor 2 (FGFR2). It is characterized by the premature fusion of cranial sutures, leading to a brachycephalic head shape, and midfacial hypoplasia. The aim of this study was to investigate the effect of the FGFR2 mutation on the microarchitecture of cranial bones at different stages of postnatal skull development, using the FGFR2^C342Y^ mouse model. Apart from craniosynostosis, this model shows cranial bone abnormalities. High‐resolution synchrotron microtomography images of the frontal and parietal bone were acquired for both FGFR2^C342Y/+^ (Crouzon, heterozygous mutant) and FGFR2^+/+^ (control, wild‐type) mice at five ages (postnatal days 1, 3, 7, 14 and 21, *n* = 6 each). Morphometric measurements were determined for cortical bone porosity: osteocyte lacunae and canals. General linear model to assess the effect of age, anatomical location and genotype was carried out for each morphometric measurement. Histological analysis was performed to validate the findings. In both groups (Crouzon and wild‐type), statistical difference in bone volume fraction, average canal volume, lacunar number density, lacunar volume density and canal volume density was found at most age points, with the frontal bone generally showing higher porosity and fewer lacunae. Frontal bone showed differences between the Crouzon and wild‐type groups in terms of lacunar morphometry (average lacunar volume, lacunar number density and lacunar volume density) with larger, less dense lacunae around the postnatal age of P7–P14. Histological analysis of bone showed marked differences in frontal bone only. These findings provide a better understanding of the pathogenesis of Crouzon syndrome and will contribute to computational models that predict postoperative changes with the aim to improve surgical outcome.

## Graphical abstract

Crouzon syndrome, caused by FGFR2 mutations, leads to premature cranial suture fusion and abnormal head shapes. This study used FGFR2C342Y mice to examine the mutation's effect on cranial bone microarchitecture during postnatal development. High‐resolution imaging of frontal and parietal bones in mutant and wild‐type mice revealed significant differences in bone volume fraction, canal and lacunar morphometry, especially in frontal bones. Mutants had larger, less dense lacunae around postnatal days 7–14. These findings improve understanding of Crouzon syndrome and aid in enhancing surgical outcomes.

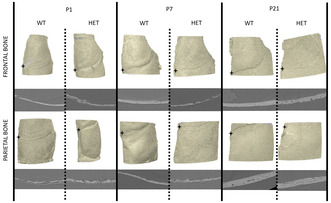

## 1 INTRODUCTION

Craniosynostosis (CS) is a medical condition with an overall prevalence of 1 in 2100 live births (Boulet et al., 2008; Lajeunie et al., 1995) and is defined as the premature closure of cranial sutures leading to abnormal calvarial growth (Governale, 2015). At birth, the sutures are normally unfused to facilitate passage of the infant's head through the birth canal (Persson, 1995). Furthermore, the patent sutures work as shock absorbers and accommodate the growth of the brain, which is estimated to triple in volume over the first year of life (Derderian & Seaward, 2012; Persson, 1995). Closure of the sutures is reported to happen naturally later in adulthood, starting from 22 to 30 years of age (Todd & Lyon, 1924; Wang et al., 2022), except for the metopic suture, which is reported to close as early as 3 months of age (Vu et al., 2001).

CS can occur as a non‐syndromic condition or as part of a characterized craniofacial syndrome, such as Apert, Crouzon, Muenke, Pfeiffer or Saethre‐Chotzen syndrome. About 15% of CS cases are syndromic, typically resulting from gain‐of‐function mutations in the FGFR gene. Crouzon syndrome, one of the most common, appears in 16.5 per million live births and involves an autosomal dominant. The most common coding mutation responsible for Crouzon syndrome, one of the most common presentation of syndromic craniosynostosis, is a point mutation in exon 9 of the FGFR2 gene, in which a cysteine molecule is substituted by a tyrosine residue (p.C342Y) and the IIIC isoform of the receptor is affected (Peskett et al., 2017). Phenotypically, this mutation leads to premature fusion of the coronal sutures and a brachycephalic head shape. Untreated CS can cause serious issues such as increased intracranial pressure (ICP), upper airway obstruction, hearing disturbances and eye problems like strabismus, ptosis and exophthalmos. Treatment often involves surgery to increase intracranial volume, lower ICP, maintain an adequate airway, achieve ideal tooth placement, and normalize skull and facial shape. However, unpredictable outcomes and incomplete correction are common in multi‐sutural or syndromic CS due to a limited understanding of skull morphology. Better insight into the underlying pathology and age‐related changes could improve surgical outcomes. (Baird et al., 2012; Cohen & Kreiborg, 1992; Flaherty et al., 2016; Gault et al., 1992; Gopal et al., 2017; Kimonis et al., 2007; Mathijssen et al., 2021; O"Hara et al., 2019; Perlyn et al., 2006; Sakamoto et al., 2016; Wilkie et al., 2017).

To gain more knowledge about the pathogenesis of this disease, the FGFR2 mutation has previously been introduced to mice (Eswarakumar et al., 2004). Mice have been used widely in biomedical research, including craniofacial biology, due to their anatomical and physiological resemblances with humans (Bryda, 2013; Lee et al., 2019). It has been found that the mouse knock‐in of the human C342Y mutation (FGFR2^C342Y^) leads to a craniofacial phenotype that closely mimics humans, where coronal CS as well as midfacial hypoplasia are present (Eswarakumar et al., 2004; Hoshino et al., 2023; Peskett et al., 2017).

The mouse model of Crouzon syndrome, FGFR2^C342Y/+^ was developed to understand the role of the FGFR2c variant (Eswarakumar et al., 2004). Multiple studies have shown that the FGFR2^C342Y/+^ mouse model has phenotypes that are mostly parallel to what is seen in human patients (Eswarakumar et al., 2004; Perlyn et al., 2006) (Perlyn et al., 2006). The FGFR2^C342Y/+^ mouse (Crouzon/mutant mouse) is characterized by a rounded skull vault (dome‐shaped), premature fusion of the cranial sutures, proptotic eyes, shortened midface, and high arched or cleft palate (Derderian & Seaward, 2012; Peskett et al., 2017). Unlike the more variable cranial suture fusions in humans, the premature fusion in the mouse model presents itself consistently with fused coronal sutures, partial fusion at the lambdoid suture and partially separable sagittal sutures (which typically remains unfused in mice) (Grova et al., 2012). Various studies have hypothesized and demonstrated that this coding mutation responsible for Crouzon syndrome leads to increased osteoblast differentiation and bone formation at early stages of development, while inhibiting these processes at a later stage (Peskett et al., 2017). Moreover, the mutation is associated with increased apoptosis and diminished bone volume and density (Eswarakumar et al., 2004; Liu, Kyung, et al., 2013). Previous work has also shown that frontal bones have different mechanical properties compared to parietal bones in FGFR2^C342Y^ mice (Moazen et al., 2015).

The aim of this cross‐sectional study was to assess the early postnatal development of the Crouzon mouse skull by means of synchrotron microtomography, to allow investigation of bone microstructure and porosity density changes at different stages of postnatal development. We hypothesized that cranial bone micromorphology is affected by the FGFR2 mutation and that such an effect is most evident in the anterior part of the skull.

### 1.1 Methods

This project was carried out using wild‐type (WT, *n* = 6) and heterozygous mutant (HET, *n* = 6) mice, respectively, carrying two normal FGFR2 alleles or one mutant with one normal FGFR2 allele. The HET mice with the targeted mutation in FGFR2 (Fgfr2^tm4Lni^ also known as Fgfr2c^C342Y^, MGI: 3053095) were originally described by Eswarakumar et al (2004). and used to mimic the clinical spectrum as seen in Crouzon patients. The mice were re‐derived through the European Mouse Mutant Archive (EMMA) at MRC Harwell (CD1.Cg‐Fgfr2^tm4Lni^, EMMA Strain ID EM:02488) and kept as heterozygous breeding pairs in the animal care facilities of University College London Biological Services, where they were maintained as previously described by Peskett et al (2017). A genotyping PCR protocol was performed to divide litters into WT or HET as previously published. All animal procedures were carried out commensurate with the UK Animals (Scientific Procedures) Act 1986 (Project License number PP8161503). Data Collection and analysis complied with the ARRIVE guidelines and were performed under the supervision of UCL Biological Services.

**FIGURE 1:**
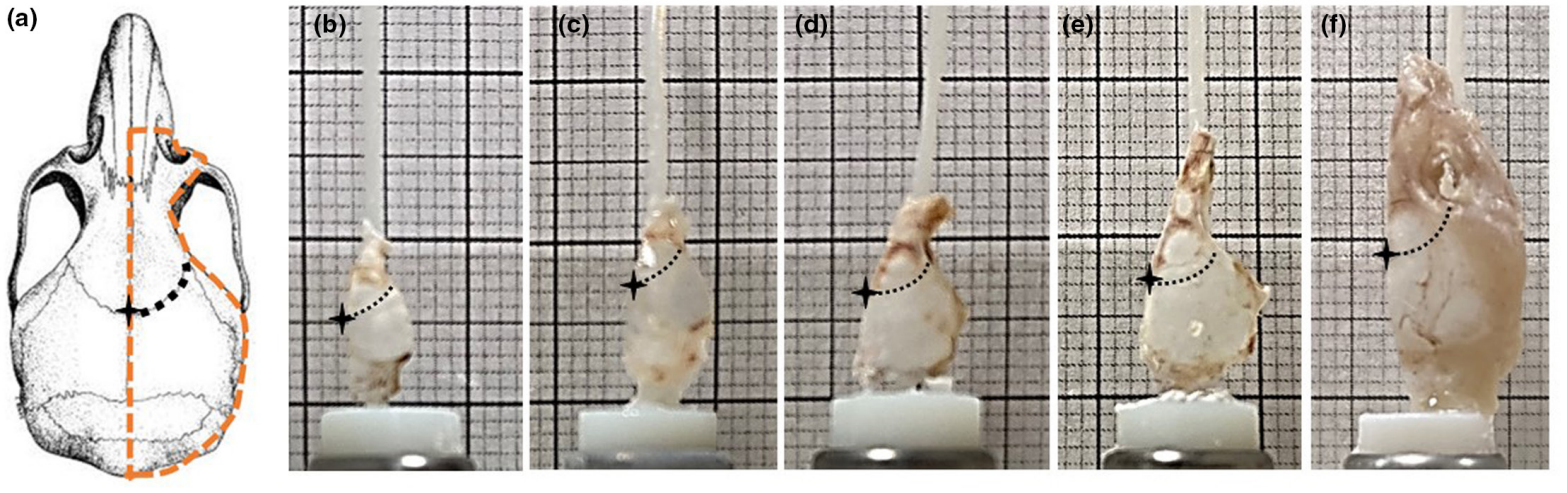
Right‐sided (highlighted on the left) mounted calvaria (WTs) at (a) P1, (b) P3, (c) P7, (d) P14 and (e) P21. The coronal suture was highlighted using a dotted black line in each picture for clarity, with the bregma identified as a star. The grid on the back has a spacing of 1 mm (thin lines) and 1 cm (thick lines).

The frontal and parietal bones from both WT and HET mice were collected at postnatal days (P) 1, 3, 7, 14 and 21. From each genotype, six frontoparietal bone samples were collected and analysed (Figure 1). To obtain the bone samples, mice were asphyxiated by exposure to rising concentrations of carbon dioxide gas. Whole‐mount skeletal preparations were then made by removing the skin and connective tissue and dissecting the calvarium. Subsequently, the dissected calvaria were washed with phosphate buffer saline (PBS) solution to detach blood and any adhering tissue. The bone samples were separated along the sagittal suture (Figure 1, resulting in a left and right sample per skull) and fixated for 48 h in 10% neutral buffered formalin.

### 1.2 Bone morphometry

Each calvarium was mounted on cryocaps and stored in cryovials at −20°C until imaged with synchrotron microtomography at the Diamond Light Source I13‐2. Only right frontoparietal bone samples were scanned, unless the sample was noticeably damaged, then the left sided samples would also be scanned (see Figure S1 for an overview of the scanned calvarial sides). Two hours before image acquisition, samples were thawed in PBS at room temperature.

Synchrotron X‐ray microtomography were obtained using a partially‐coherent, filtered, polychromatic ‘pink’ beam (8–30 keV) of parallel geometry generated by an undulator of 5 mm gap. For each scan, 4000 projection images of exposure time were acquired at equally‐spaced angles over 180 degrees of continuous rotation around the sagittal (vertical) axis of the sample. The last projection was compared to the first to check for possible sample deformation, bulk movements and radiation damages. Images were collected with a pco.edge Camera Link 5.5 (PCO AG, Germany) detector mounted on a visual light microscope for variable magnification. Samples were placed as close as possible to the detector, and 1.25× objective lens (pixel size 2.6 μm, field of view 6.7 mm × 5.6 mm, exposure time 0.15 s) was firstly used to capture the full morphology of half calvaria. Afterwards, a 2× objective lens (pixel size 1.625 μm, field of view 4.2 mm × 3.5 mm, exposure time 0.04 s) was used to create a magnified view of either frontal or parietal bone sections, separately. Image projections were flat‐ and dark‐field corrected, and ring artefact suppression and optical distortion correction were applied using the Savu framework (Atwood et al., 2015).

**FIGURE 2:**
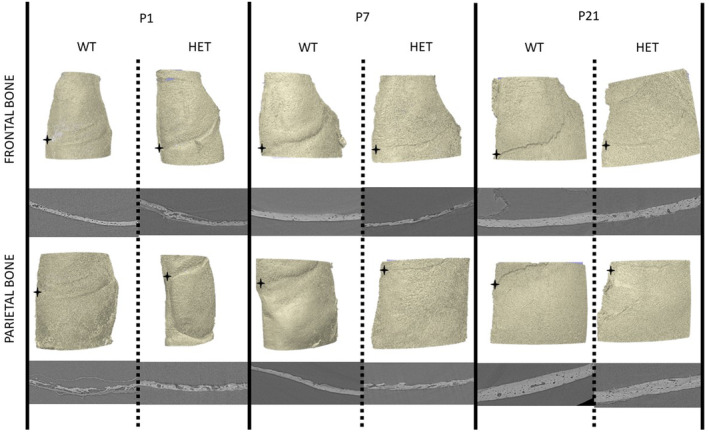
Samples of 3D reconstruction of frontal (top row) and parietal (bottom row) bones of representative WT (on the left in each Figure pair) and HET (on the right in each Figure pair) of P1, P7 and P21 mouse samples. Below each reconstruction, a sample of synchrotron CT cross section.

Fiji software (ImageJ version 1.53n) was used to convert the obtained synchrotron data sets from 16‐bit to 8‐bit to conduct the preliminary processing using Simpleware (version Scan IP O‐2018.12‐SP2), where the images were first aligned in a similar position. As visible in Figure 2, the skulls were aligned in a way that the bregma (the anatomical point on the skull where the coronal and sagittal suture intersect) and the medial parts of the frontal and parietal bone were positioned parallel to the *xz*‐plane. Following the alignment of all skulls in the same orientation, Volumes of Interests (VOIs) were selected. A method like that reported by Liu et al (2013). was adopted to define the VOIs. To do this, the user manually selected the location of the bregma. Next, regular spaced planes of 0.5 mm length, 0.5 mm width and depth corresponding to the bone thickness, were selected on pre‐defined positions. The first VOI (VOI1) was identified 0.5 mm away from the bregma in both the *x*‐axis and *z*‐axis (along respectively the coronal and sagittal suture). The second VOI (VOI2) was selected at a 0.2 mm distance away from VOI1 in the *x*‐axis and the third VOI (VOI3) at a 0.2 mm distance away from VOI1 in the *z*‐axis. A total of 360 VOIs have been analysed in this study (60 samples × 3 frontal VOIs + 3 parietal VOIs).

These VOIs were uploaded in CT Analyser (SkyScan software 1.18.8.0), where subsequently, suitable thresholds were chosen to create binary images of the intracortical pores and standard morphometric measurements of interests were quantified. Bone porosity was assessed within bones. Thus, canals and osteocyte lacunae were segmented and analysed as previously reported in the literature (Table 1) (Bach‐Gansmo et al., 2016; Carriero et al., 2014; de Paolis et al., 2021; McCreadie et al., 2004; Wittig et al., 2016). At the tissue level, we analysed tissue volume (Ct.TV), cortical bone volume (Ct.BV), cortical thickness (Ct.Th), bone volume fraction (Ct.BV/Ct.TV), canal number (N.Ca), canal total volume (Ca.V), canal number density (N.Ca/Ct.TV), canal volume density (Ca.V/Ct.TV) and mean canal volume (<Ca.V>). At the cellular level, the number of osteocyte lacunae (N.Lc), lacunar total volume (Lc.V), lacuna number density (N.Lc/Ct.TV), lacuna volume density (Lc.V/Ct.TV) and mean lacuna volume (<Lc.V>) were examined.

**TABLE 1:** Summary of the bone morphometric measurement data collected as part of this study.

Morphometric measurement	Symbol	Unit
Total tissue volume	Ct.TV	μm^3^
Cortical bone volume	Ct.BV	μm^3^
Bone volume fraction	Ct.BV/Ct.TV^a^	%
Cross‐sectional thickness	Ct.Th^a^	μm
Number of canals	N.Ca	–
Canals total volume	Ca.V	μm^3^
Number of lacunae	N.Lc	–
Lacunar total volume	Lc.V	μm^3^
Average canal volume	<Ca.V>^a^	μm^3^
Average lacunar volume	<Lc.V>^a^	μm^3^
Lacunar number density	N.Lc/Ct.TV^a^	mm^−3^
Lacunar volume density	Lc.V/Ct.TV^a^	%
Canal number density	N.Ca/Ct.TV^a^	mm^−3^
Canal volume density	Ca.V/Ct.TV^a^	%

^a^ These were assessed by means of a general linear model.

Statistical analysis was performed using MATLAB comparing Ct.BV/Ct.TV, Ct.Th, N.Ca/Ct.TV, Ca.V/Ct.TV, <Ca.V>, N. Lc/Ct.TV, Lc.V/Ct.TV and <Lc.V> between the different groups of mice organized by age, anatomical location (parietal and frontal) and mouse strain (WT and HET). General linear model analysis was performed for each of these variables to assess the effect of age, anatomical location, genotype and their interactions.

### 1.3 Bone histology

To explain the morphological changes observed between WT and HET calvarial bones, we analysed frontal and parietal by histological staining. The skulls of a separate set of Fgfr2^C342Y/+^ and control mice were dissected and fixed in 4% PFA, then demineralized in 10% EDTA solution. The samples were embedded in paraffin after dehydration and sagittal sections were obtained at 7–8 μm thickness, and areas of bone on both sides of the coronal suture representing the frontal and parietal bones were imaged. The stained slides were cleared by xylene and applied to the mounting agent for observation.

## 2 RESULTS

### 2.1 Bone morphometry

**FIGURE 3:**
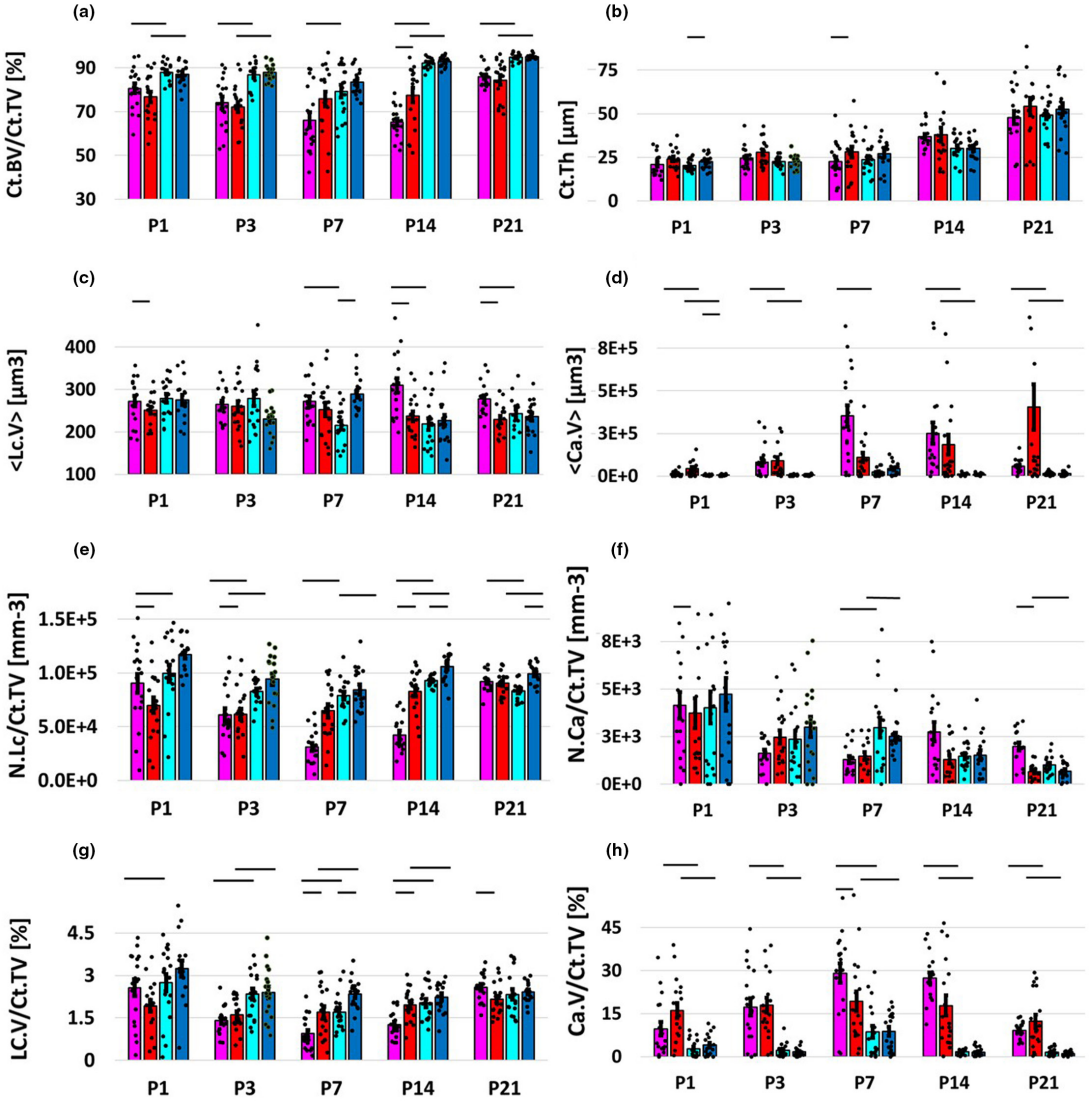
Scatter bar plots (mean ±SE) showing variation with age of bone volume fraction (Ct.BV/Ct.TV; a), cross‐sectional thickness (Ct.Th; b), average lacunar volume (<Lc. V>; c), average canal volume (<Ca.V>; d), lacunar number density (N.Lc/Ct.TV; e), canal number density (N.Ca/Ct.TV; f), lacunar volume density (Lc.V/Ct.TV; g) and canal volume density (Ca.V/Ct.TV; h). WT parietal bone in blue, HET parietal bone in cyan, WT frontal bone in red, HET frontal bone in magenta; statistical differences are indicated with straight lines over bars (*p* < 0.05).

Strain differences between frontal and parietal bone were relatively consistent throughout the measurements, with the parietal bone having a statistically higher bone Ct.BV/Ct.TV, lower <Lc.V>, lower <Ca.V>, higher N.LC/Ct.TV, higher Lc.V/Ct.TV and lower Ca.V/Ct.TV at most time points (Figure 3). Ct.Th was not different between frontal and parietal bone within the same strain, at any time point. Analysis of the differences between the two genotypes showed how the mutant (HET) bones, in particular the frontal bone, grow differently in some aspects. Average lacunar volume is statistically higher in the HET group at 1, P14 and P21 (Figure 3c). Lacunar number density is significantly lower in HET frontal bone for P1, P3 and P14 (Figure 3e). A similar trend was visible for lacunar volume density (Figure 3g), where statistical difference was found at P3, P14 and P21. This provides evidence that the FGFR2 mutation reduces or delays bone formation, specifically in the frontal bone (Robling & Bonewald, 2020; Wang et al., 2021). Canal morphometry showed statistical differences in N.Ca/Ct.TV at P3 and P21 for the frontal bone and in Ca.V/Ct.TV at P3, also for the frontal bone.

General linear model results are reported in Table 2. Age had statistically significant effect only on Ct.BV/CT.TV, while for other parameters age effect was modulated by either anatomical location (<Ca.V>, Lc.V/Ct.V) or genotype (<Ca.V>, <Lc.V>). Anatomical location had a statistical effect on Ct.BV/CT.TV, Ca.V/Ct.TV, N.Lc/Ct.TV, Lc.V/Ct.TV.

**TABLE 2:** General linear model for the morphometric parameters.

General linear model						
	Intercept	Age	Bone (front/par)	Genotype (WT/HET)	Age × bone	Age × genotype
Ct.BV/Ct.TV	72.05	**0.28** *	**11.71** *	1.40	0.07	0.12
Cs.th	20.80	**1.19** *	−3.60	1.76	0.078	0.14
<Lc.V>	267.03	0.68	−0.83	4.23	−1.38	**−1.95** *
<Ca.V> × 103	166.03	7.90	−100.31	−79.08	**−15.27** *	**14.53** *
N.Lc/Ct.TV × 103	59.80	0.54	**27.92** *	4.264	−0.60	0.58
N.Ca/Ct.TV × 103	2.87	41.41	0.92	0.89	−0.077	−0.11
Lc.V/Ct.TV	1.63	0.02	**0.79** *	0.14	**−0.03** *	0.0002
Ca.V/Ct.TV	19.18	−0.09	**−13.61** *	−0.89	−0.01	−0.06

* *p* < 0.05 (in bold).

### 2.2 Bone histology

**FIGURE 4:**
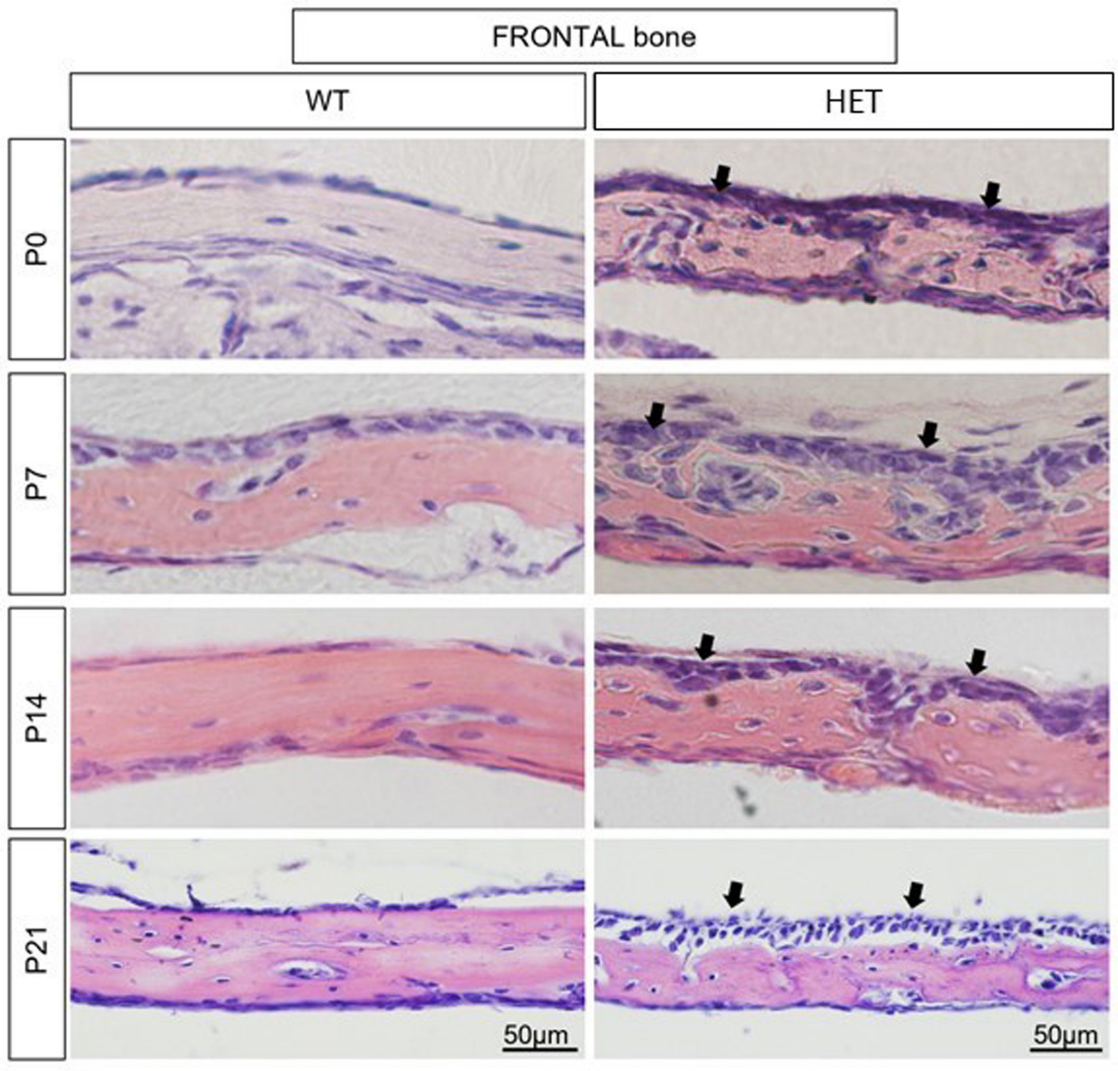
H&E‐stained frontal bone of wild‐type (WT) and mutant (HET: Crouzon) mouse at postnatal days P0, P7, P14 and P21. Mutant (Fgfr2‐C342Y) frontal bone has more periosteal cells (arrows) and a smaller bone matrix (pink staining), resembling reduced ossification at all stages. Scale bar = 50 μm.

Frontal bones stained with Haemotoxylin and Eosin were markedly different in HET mice compared to WT controls. Overall, bone looked less mature, with lower levels of ossification and higher numbers of osteogenic cells, mainly in the periosteal layer (Figure 4). Histologically, WT and HET parietal bones were not showing any differences (Figure S1).

## 3 DISCUSSION

In this study, a mouse model for Crouzon syndrome was used to identify the specific effects of FGFR2 mutation on calvarial bone microstructure. During normal craniofacial skeletal development, the calvarial bones are formed by intramembranous ossification. Intramembranous ossification, as well as the formation and maintenance of the cranial sutures, are precisely controlled processes and any perturbation may induce abnormal cranial bone formation or impaired suture fusion (Marie et al., 2008). Previous research has found that cells of the osteoblastic lineage are reliant on the expression and activity of FGFRs, which are known to activate several pathways that control proliferation, differentiation and apoptosis of bone tissue (Dailey et al., 2005; Ornitz & Marie, 2015). Furthermore, the FGF‐FGFR signalling cascade has been found to be involved in the suture homeostasis (Teven et al., 2014). As mentioned, the p.C342Y mutation in the FGFR2 is the most common coding mutation causing Crouzon syndrome. Several studies have been performed to further elucidate the hypothesis that Crouzon syndrome associated with this mutation leads to intrinsic alterations in osteoblast differentiation and bone formation.

The results from our study support and supplement previous findings. Our measurements show differences between HET and WT in frontal bone morphology at most time points, with particular difference in lacunar related variables (<Lc.V>, N.Lc/Ct.TV, Lc.V/Ct.TV) with larger, less dense lacunae around the age of P7–P14. Eswarakumar et al (2004). found a significantly higher expression of Spp1, a bone matrix protein released by osteoblasts, and significantly increased number of osteoblast progenitor cells during early embryonic development in HET cranial bones, while the number of osteoblasts seemed to be stabilized or possibly decreased at postnatal day 1. Instead of examining osteoblasts, here we measured for the first time the number and volume densities of osteocyte lacunae between FGFR2 WT and HET mice at several postnatal time points as a marker of bone development. Lacunae are small cavities in the bone containing an osteocyte, which was originally an osteoblast that was inglobated upon formation of the bone matrix (Robling & Bonewald, 2020). A lower number of osteocyte lacunae is likely linked to a lower number of osteoblasts producing matrix; therefore, the number of lacunae can serve as an appropriate marker for bone development. Lacunar number density in HET compared to WT was found statistically lower in the frontal bone at P1, P3 and P14 and in the parietal bone at P14 and P21 (Figure 3e). Lacunar volume density in HET compared to WT was found statistically lower in P3, P14 and P21 and in the parietal bone at P3 (Figure 3g). Full suture closure has been reported to happen typically around P21; therefore, it is reasonable that an effect on bone micromorphology is evident before this time. Considering that FGF and ERK signalling is important for osteocyte differentiation (Kyono et al., 2012), the current evaluation of the effect of the FGFR2 mutation on osteocytes is very relevant to further understand bone biology and function.

Our data showed that bone volume fraction (Ct.BV/Ct.TV) was lower in the frontal bone at P14 (Figure 3a), which agrees with the results of Liu et al (2013). who demonstrated that FGFR2^C342Y/+^ mice have significantly diminished bone volume in frontal bones, and that several osteoblastic marker genes, such as Runx2, TNAP and BSP, were normal or enhanced in early differentiating stages and diminished during later stages (from postnatal day 12 and later). The same decrease in bone volume was found in other studies researching FGFR‐associated CS (2013) and in other mouse models, such as FGFR2^P244R/+^ and FGFR2^S250W/+^, mimicking Muenke and Apert syndrome, which showed a diminished bone volume and/or bone formation in comparison to the WT mice (Chen et al., 2003; Twigg et al., 2009).

Recent evidence (Wang et al., 2021) has linked osteocytes with direct matrix formation and therefore a lower osteocyte density would be directly linked to impaired bone formation. General linear model showed that while overall bone porosity (related to Ct.BV/Ct.TV) is affected by age, embryonic origin (frontal vs. parietal bone) had effect on lacunar number density, lacunar volume density and canal volume density, with average lacunar and canal volume being affected also by the interaction between age and genotype.

In our results, the canal volume density is statistically higher (Figure 3h **)** in the HET frontal bone compared to WT at P3 and the canal number density (Figure 3f) is higher at both P3 and P21. The FGF pathways, and particularly FGF‐1 and FGF‐2, are well known to play a role in angiogenesis (Friesel & Maciag, 1995). Although FGFs are potent angiogenic factors, their precise roles have not been fully understood and possibly they indirectly control neo‐vascularization collectively with other growth factors (Murakami & Simons, 2008). Notably, Tholpady et al (2004). found a significantly higher blood vessels diameter in calvarial bone from Crouzon patients compared to normal calvarial bone. Our and their study therefore suggest that the mutation of the FGFR2 does influence the angiogenesis and results in a more fragile bone in Crouzon patients.

The main hypothesis that has been tested during this study was if the effect of the FGFR2 mutation would be more apparent in frontal bone than in parietal bone. Both bones have different embryonic origins; whereas the frontal bone is neural crest derived, the parietal bone has a mesodermal origin (Jiang et al., 2002). Our data support these the evidence that frontal and parietal bones indeed are affected differently by FGFR2 mutation, with the frontal bone in HET mice exhibiting higher porosity (mostly at P14) due to a higher canal volume density and a lower lacunar volume and number density, which may be linked to impaired bone deposition capability. Liu et al (2013). found that the frontal bone of HET mice encountered increased apoptosis and decreased bone mineralization in comparison to their parietal bone, which suggests that the frontal bone cells might be more prone to the effects of the FGFR2 mutation. Previous studies in healthy mice have found that frontal bone‐derived osteoblasts experience a greater proliferation rate, higher osteogenic potential and increased activation of FGF pathways (Li et al., 2010). Moreover, Quarto et al (2009). illustrated that the frontal bones of wild‐type mice experience an upregulated expression of FGFs and FGFRs in comparison to the parietal bones. These two observations may contribute to the theory that the innate differences between frontal and parietal bone result in the abnormalities caused by the FGFR2 mutation to be more apparent in frontal than parietal bone, since frontal bone has a higher FGF‐signalling competence. Using in vitro culture approaches, Doro et al. found that neural crest‐derived osteoblasts readily generate bony nodules, while mesodermal osteoblasts do so less efficiently (Doro et al., 2019).

In this study, the early postnatal development of the Crouzon Skull was quantitatively assessed: the results revealed that the bone microarchitecture of calvarial bone samples in Fgfr2^C342Y/+^ mice are different from WT control mice and that the FGFR2 mutation leads to morphological abnormalities at the microlevel that have not been reported before. As hypothesized, the significant difference between frontal and parietal bone was confirmed and corroborates the idea that genetic and cell biology changes are reflected in bone microarchitecture alterations. These observations increase our understanding of the pathogenesis of syndromic craniosynostosis and will inform clinical decision making, since calvarial bone quality is often considered during preoperative planning of complex craniosynostosis procedures.

## AUTHOR CONTRIBUTIONS

EP and AB conceived and designed the project. SA, ZvdD, JH, DS, MC, MS and Y‐MC acquired, analysed and interpreted data. SA, ZvdD, AC, EP and AB wrote the paper. All authors commented on and contributed to the paper.

## Supporting information


Figure S1.

## Data Availability

Research data are not shared.
