# Supplementary material for: Cranial bone microarchitecture in a mouse model for syndromic craniosynostosis
Source: J Anat. 2024 Aug 2;245(6):864–73. doi: 10.1111/joa.14121 (PMC11547221; doi:10.1111/joa.14121)
Supplement: Supplementary file 1 — Figure S1. [file JOA-245-864-s001.docx]

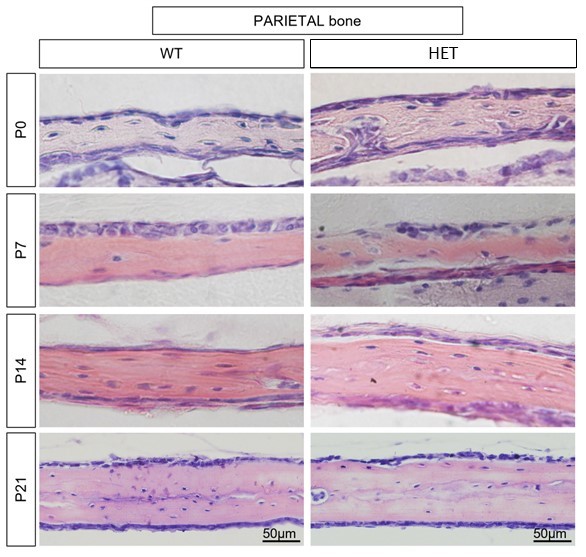


**Supplementary Figure 1**: *H&E-stained parietal bone of wild-type (WT) and mutant (HET: Crouzon) mouse at postnatal day P0, P7, P14 and P21. No major differences can be observed. Scale bar = 50μm*
